# Post-Translational Modification and Subcellular Compartmentalization: Emerging Concepts on the Regulation and Physiopathological Relevance of RhoGTPases

**DOI:** 10.3390/cells10081990

**Published:** 2021-08-05

**Authors:** Inmaculada Navarro-Lérida, Miguel Sánchez-Álvarez, Miguel Ángel del Pozo

**Affiliations:** 1 Centro de Biología Molecular “Severo Ochoa”, Department of Molecular Biology, Campus de Cantoblanco, School of Sciences, Universidad Autónoma de Madrid, 28049 Madrid, Spain; 2 Centro Nacional de Investigaciones Cardiovasculares (CNIC), Mechanoadaptation and Caveolae Biology Lab., Cell and Developmental Biology Area, 28029 Madrid, Spain; msancheza@cnic.es

**Keywords:** Rho GTPases, post-translational modifications (PTMs), subcellular compartmentalization, plasma membrane (PM), cytoskeleton, nucleus, cell mechanoadaptation, disease

## Abstract

Cells and tissues are continuously exposed to both chemical and physical stimuli and dynamically adapt and respond to this variety of external cues to ensure cellular homeostasis, regulated development and tissue-specific differentiation. Alterations of these pathways promote disease progression—a prominent example being cancer. Rho GTPases are key regulators of the remodeling of cytoskeleton and cell membranes and their coordination and integration with different biological processes, including cell polarization and motility, as well as other signaling networks such as growth signaling and proliferation. Apart from the control of GTP–GDP cycling, Rho GTPase activity is spatially and temporally regulated by post-translation modifications (PTMs) and their assembly onto specific protein complexes, which determine their controlled activity at distinct cellular compartments. Although Rho GTPases were traditionally conceived as targeted from the cytosol to the plasma membrane to exert their activity, recent research demonstrates that active pools of different Rho GTPases also localize to endomembranes and the nucleus. In this review, we discuss how PTM-driven modulation of Rho GTPases provides a versatile mechanism for their compartmentalization and functional regulation. Understanding how the subcellular sorting of active small GTPase pools occurs and what its functional significance is could reveal novel therapeutic opportunities.

## 1. Introduction

Cell motility is essential for tissue development and homeostasis in multicellular organisms, but its dysregulation plays a crucial role in pathological processes such as tumor invasion and metastasis. The actin cytoskeleton is the engine that drives polarized cell behavior through the spatial and temporal control of cell protrusiveness, adhesion, contractility and rear retraction. Changes in the actin cytoskeleton modulate not only focal adhesion dynamics and cell contraction but also the physical properties (i.e., rigidity, compliance) of the cell, including its nucleus. Characterizing how these cellular systems function in an integrated manner is essential to understand cell migration. The ability of tumor cells (TCs) to actively migrate through tissue structures and colonize both adjacent and distant sites is intensively studied because metastatic disease is by far the prime death cause in cancer patients [1,2]. Additionally, the communication between TCs and the tumor microenvironment (TME) is an important aspect of both tumor progression and metastasis, but we have a very limited understanding as to how it occurs. A key emerging aspect is the adaptation of TCs to changes in stromal extracellular matrix (ECM). A highly dynamic structure, the ECM undergoes biochemical and physical remodeling in response to different cues, mainly exerted by altered populations of activated fibroblasts (cancer-associated fibroblasts or CAFs). Actomyosin contraction and cell orientation are pivotal aspects of this reciprocal crosstalk between CAFs and ECM [3,4]. Stromal ECM remodeling, in turn, drives TC morphological organization and signaling rewiring to foster migration and metastasis. These adaptations are crucial to both promote TC motility as well as to enable them to squeeze through tight interstitial spaces. Indeed, the architecture of the ECM also determines TC metastatic success by imposing a physical barrier to the scape of TCs from the primary tumor site. During this process, networks regulating cell morphology and size and parameters associated with the extracellular environment are integrated to determine TC aggressiveness and metastatic ability. The nucleus has recently received increased attention regarding TC metastasis because it is the largest and stiffest structure in the cell, constituting the main limiting factor for cellular deformation during TC migration. Conversely, changes in nuclear morphology and deformability can also have a direct impact on TC behavior through the altered integrity of the nuclear envelope (NE) and the genomic material [5].

The dynamic architecture of the actin cytoskeleton is a key parameter in these complex processes [6]. The basic molecular machinery underlying the assembly and disassembly of actin filaments consists of a variety of actin-binding proteins that regulate the dynamic behavior of the cytoskeleton in response to different signals. Rho small GTPases act as master molecular switches regulating cytoskeletal remodeling [7]. ‘Classic’ models depict active Rho GTPases proteins as residing and functioning predominantly at the plasma membrane (PM), but recent research supports different pools of active Rho GTPases can also operate in intracellular compartments, including the cell nucleus. How is this functional segregation regulated? Despite the fact that multiple mechanisms can intervene in Rho GTPase regulation, the dynamic modulation of RhoGTPases is critically dependent on their post-translational modifications. Lipid modifications control protein localization and activity. The functional dependence on prenylation and palmitoylation of different Rho GTPases suggests that PTMs could critically control their subcellular trafficking across spatially distant compartments—such as the PM and the nucleus—and their temporal regulation, allowing for the bidirectional transfer of information between them. Here, we discuss recent advances in our understanding of the post-translational regulation and subcellular compartmentalization of RhoGTPases and their functional relevance for enabling cell morphology control required during cell migration through 3D microenvironments. A better understanding of the mechanisms underlying the crosstalk between mechanical stimuli and biochemical responses through specific subcellular compartmentalization of RhoGTPases could help us to identify novel therapeutic targets against cancer disease.

## 2. General Aspects of Rho GTPase Modulation

The Rho family of proteins make up a major branch of the Ras superfamily of small GTPases. To date, 22 human genes encoding at least 25 proteins were described, the best characterized ‘classical’ members being RhoA, Rac1 and Cdc42. They share a high degree of amino acid sequence homology, indicating high biological relevance and selective pressure, especially regarding residues directly involved in the binding and hydrolysis of guanine nucleotides [8,9].

**Figure 1:**
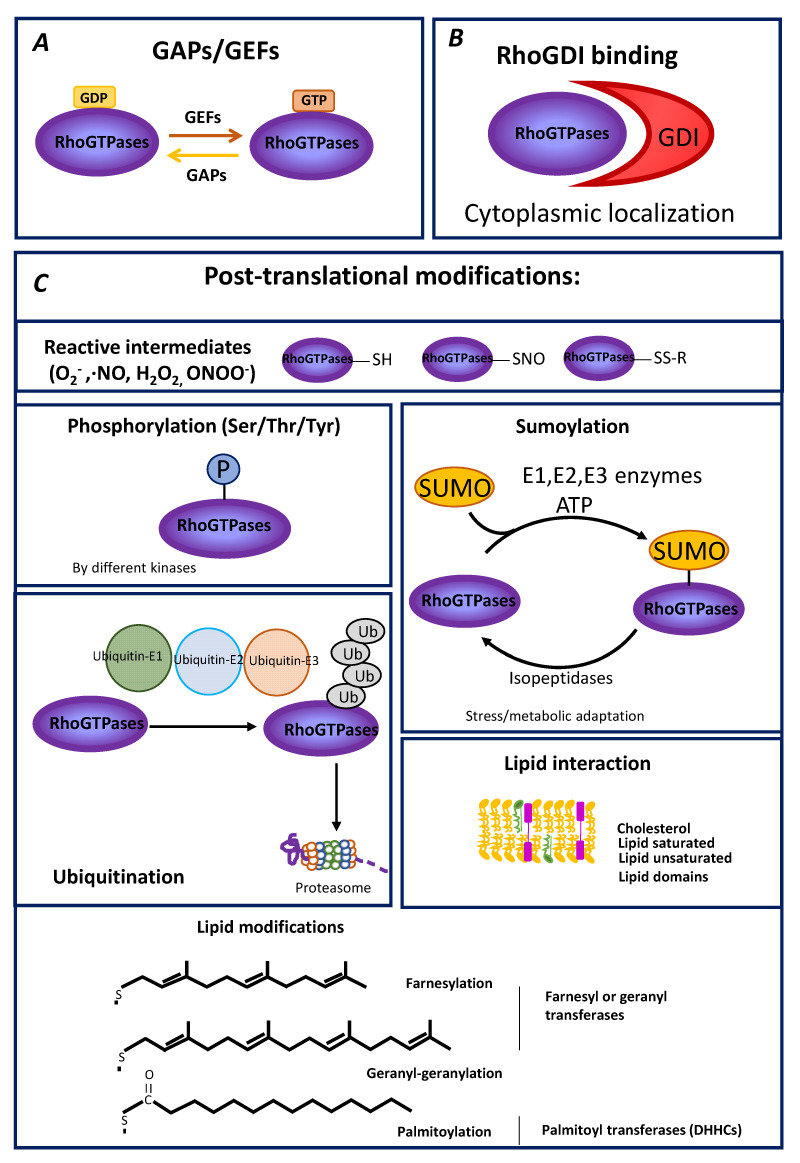
Mechanisms forRho GTPase modulation. (**A**,**B**) GAPs and GEFs (**A**) and GDI (**B**) constitute primary regulation layers controlling GTPase activity through the dynamics of GDP–GTP cycling and the sequestration from membrane environments, respectively. (**C**) Different types of non-mutually exclusive post-translational modifications modulating small GTPase localization and/or activity are summarized. (SUMO: Small Ubiquitin-like Modifiers).

As molecular switches, Rho small GTPases cycle between an active GTP-bound form and an inactive GDP-bound state, and mutations in positions 12 or 61 (usually Gly12Val or Gln61Leu) result in catalytically deficient Rho proteins, thus considered to drive constitutive activation of downstream signaling. The control of the balance between the GTP-bound active state and the GDP-bound inactive state constitutes a first essential regulatory layer onto which several mechanisms converge [10,11]. Guanine nucleotide exchange factors (GEFs) promote GDP-to-GTP exchange, leading to GTPase activation. Conversely, GTPase-Activating Proteins (GAPs) stimulate GTPase activity, favoring an inactive state. Thus, GAPs and GEFs can be considered negative and positive regulators of small GTPases, respectively [12,13]. Mutations in position 17 (commonly Thr17Asn) exhibit reduced nucleotide binding and were thus described as dominant-negative forms of these GTPases, presumably through sequestration of RhoGEFs [14,15]. However, this model requires further investigation because while many RhoGEFs can control different Rho GTPases, the expression of one mutant small GTPase member does not always inhibit the signaling mediated by other small GTPases (Figure 1).

Additional studies based on the characterization of substrate specificities, interactomes and localization revealed at systems-level how GEFs and GAPs contextualize and spatiotemporally control Rho signaling to provide novel aspects about the emergent organization principles of Rho signaling and establishing a model by which GEFs and GAPs provide positional information based on the placement of the enzymes on dedicated cellular structures and the assembly of additional signaling network components [16].

Detailed studies based on structural and mutational analysis identified a series of regions and amino acid residues within amino-terminal regions—termed switch I and II—in RhoGTPases as essential for the exchange between the GDP- and GTP-bound states and also for efficient interaction with multiple effectors, GEFS and GAPs. However, several observations indicate that sequences outside the effector domain might also be participating in effector-mediated functions. The importance of sequences beyond the core effector domain in effector activation is exemplified by mutagenesis analysis of activated Rac1. While some residues are conserved in Rho, Rac and Cdc42 (but not Ras) proteins (Arg66, Ser71, Asp76 and Glu100), other Rac1 residues are neither conserved in Ras nor in other Rho GTPases (Asn52, Glu148, Arg163 and Cys178). These residues are surface-exposed residues and, consequently, logical candidates for involvement in effector interactions. The differential impairment in Rac1 signaling, actin reorganization and transformation exhibited by these mutants support the contribution of these residues to interaction(s) with different effectors [17].

A second mechanism regulating Rho GTPase activation state is driven by Guanine nucleotide Dissociation Inhibitors (GDIs). This family of regulatory factors controls Rho signaling through different mechanisms. Many GDIs bind the inactive, GDP-bound form of Rho-family signaling, impairing GTP exchange by GEFs and maintaining GTPases in an inactive state [18]. However, recent studies demonstrate that some RhoGDIs can also associate with GTP-bound Rho-GTPase, and the functional relevance of this association is not well understood. In the case of Cdc42, the affinity of the GDI for GTP- and GDP-bound forms is identical [19]. This capacity probably can be extended to other Rho proteins considering that the affinity relies in part on the extensive contacts between the regulatory arm of the GDI and the switch II of the GTPase, which is accessible in both states. The second and most general activity of GDIs is their ability to solubilize GTPases from cellular membranes [20].

RhoGDIs not only maintain small GTPases in their inactive GDP-bound form but also act as chaperones for small GTPases protecting them from degradation. A recent systematic study shows that the three different RhoGDIs (1, 2 and 3) interact with all possible Rho family small GTPases raising the possibility of a differential functional modulation depending on the RhoGDI binding isoform [21]. Mechanisms other than GTP–GDP cycling and GDI binding also modulate Rho GTPase signaling, including transcriptional and post-transcriptional expression regulation [22,23] (Figure 1).

The third and perhaps most complex mechanism of modulation of GTPases is through subcellular compartmentalization. A large body of literature considers most signaling initiated by Rho GTPases occurs at or around the PM; however, it is becoming clear that functionally active Rho GTPases, their GEFs and GAPs, and their effectors can be localized to different intracellular compartments beyond the PM. Despite the potential relevance of these novel intracellular pools, little is known about the mechanisms underlying its modulation.

Post-translational modifications of small GTPases are an additional regulatory layer that could integrate functional modulation and subcellular localization for the tight spatiotemporal control of GTPase activity. A wide range of PTMs was reported for small GPTases, including phosphorylation, ubiquitylation, SUMOylation and lipid modifications [24,25] (Table 1), which must be tightly controlled to ensure appropriate GTPase signaling. Moreover, it is increasingly unclear to which extent PM microdomain formation explains the regulatory impact of lipids on small GTPases and signaling lipid species and fatty acid modification seem to interplay with specific hydrophobic motifs in these proteins to achieve both reversible and irreversible modulation (Figure 1).

## 3. PTM of RhoGTPases

### 3.1. Modification of Rho GTPases by Oxidizing Species

Different cell metabolic activities producing oxidizing molecular species can be sources of oxidative stress upon dysregulation. Elevated levels of oxidizing Reactive Oxygen and Nitrogen Species (ROS and RNS) damage different cell structures such as DNA and membrane lipids and contribute to several pathological processes, including cancer [26,27]. Thiol oxidation by reactive species such as nitrogen dioxide (NO_2_), superoxide (O^−2^), hydrogen peroxide (H_2_O_2_) and peroxynitrite (ONOO^−^) can alter protein activity [28]. In contrast to lipidation, thiol oxidation of exposed cysteines can occur in the absence of enzymatic catalysis [29].

Several Ras superfamily small GTPases are redox-sensitive, and consensus acceptor motifs (NKCD, GXXXXGK(S/T)C and CGNKXD) were identified. The impact of redox agents on these redox-sensitive GTPases is similar to that of GEFs, in that they perturb GTPase nucleotide binding interactions resulting in the enhancement of guanine nucleotide exchange [30,31]. Some RhoGTPases contain a redox-sensitive thiol (Cys18 in Rac1 and Cdc42; Cys20 in RhoA), which makes direct contact with the bound guanosine nucleotide. Modification of these cysteines promotes guanine nucleotide exchange *in vitro* [32], and these Rho GTPases can also be activated upon exogenous addition of peroxide [33].

Importantly, small GTPases can also modulate the production of ROS, which can be considered part of their downstream effectors. For example, NADPH oxidases are prominent sources of ROS and affect redox signaling [34,35]. Rac1 localizes to the mitochondria [36], suggesting a role for this small GTPase in the control of a ROS-driven signaling network involving NADPH oxidases and the mitochondria. Furthermore, cellular redox state is coupled to actin cytoskeleton dynamics [37]: for example, changes in redox signaling downstream different small GTPases can downregulate RhoA activity and stress fiber formation through Rac1 because tumor cells are almost invariably subjected to different sources of oxidative stress, such as altered metabolism and mitochondrial function or hypoxia, which has a profound impact on different aspects of tumor progression; therefore, a better understanding of this regulatory layer is warranted.

### 3.2. Phosphorylation of Rho GTPases

The substantial evolutionary conservation of several phosphorylation acceptor residues in Rho GTPases supports their potential general role as modulation switches. RhoA was the first Rho GTPase shown to be phosphorylated. Cyclic AMP (cAMP)-dependent protein kinase (PKA) and cGMP-dependent protein kinase (PKG) phosphorylate RhoA on serine 188 both *in vitro* and *in vivo*. This phosphorylation does not modify its GTPase activity, nor its interaction with GEFs or GAPs, but significantly increases its interaction with RhoGDI [38,39,40]. Phosphorylation of the conserved tyrosine residue at the switch 2 region occurs in RhoA (Tyr66), Rac1 (Tyr64) [41] and Cdc42 (Tyr64) [42]. Epidermal growth factor (EGF)-mediated phosphorylation of Tyr64 on Cdc42 is mediated by the non-receptor tyrosine kinase Src and enhances the binding of active Cdc42 to RhoGDI. Binding to RhoGDI facilitates the release of active Cdc42 from the membrane and its movement between different cellular locations. Rac1 is also phosphorylated at Tyr64 by Src and focal adhesion kinase (FAK) *in vitro*. Overexpression of a Rac1-Tyr64Glu mutant (a phosphomimetic construct) suggests that phosphorylation at Tyr64 on Rac1 negatively regulates cell spreading, focal adhesion localization and its binding to GTP, RhoGDI, GEFs and its effector protein p21-activated kinase (PAK) [41].

Rho GTPases are also substrates for serine/threonine phosphorylation. RhoA is phosphorylated on Ser26 by Mst3 (mammalian Ste20-like protein kinase 3), which leads to an inactive RhoA status [43]. Rac1 is phosphorylated on Ser71 by the serine/threonine kinase protein kinase B (also known as AKT) to inhibit GTP binding [44,45], similar to the Cdc42 Ser71 phosphorylation. This PTM reduces Rac1 GTP affinity without any significant change in GTPase activity. However, both GTP-binding and GTPase activities of the Rac1 Ser71Ala point mutant are abolished, regardless of Akt activation status [45]. Rac1 Ser71 phosphorylation represents a reversible mechanism to determine the binding specificity of Rac1/Cdc42 to their downstream substrates [44] and mediates the interaction between Rac1 and 14-3-3 proteins [46,47]. This can have varied downstream effects on different processes: for example, Rac1Ser71 phosphorylation decreases its sensitivity to *Clostridium difficile* toxin A (TcdA), a potent inactivator of Ras superfamily GTPases, through irreversible glycosylation [48].

Rac1 Thr108 phosphorylation by an extracellular signal-regulated kinase (ERK) in response to EGF was also reported, leading to decreased Rac1 activity, partially through intervening Rac1 interaction with phospholipase C-γ1 (PLC-γ1). Of note, this is the only phosphorylation site reported to be directly involved in Rac1 nuclear localization [49].

The impact on the subcellular compartmentalization of small GTPases was characterized for several of these modifications. Phosphorylation of cargoes shuttling between nucleus and cytoplasm is a prominent regulatory mechanism controlling gene expression, cell growth and proliferation. Phosphorylation and dephosphorylation modulate trafficking in a cargo-specific manner, and at present, it can be difficult to predict how a phosphorylation event affects the nucleocytoplasmic trafficking of a given protein [50,51].

### 3.3. Ubiquitylation and SUMOylation

Ubiquitylation, the covalent attachment of ubiquitin to Lys residues in a target protein, can lead to degradation of the substrate or the modulation of its subcellular compartmentalization and/or activity [52]. Several Rho GTPases undergo ubiquitylation, including RhoA, Rac1, Rac1b, Cdc42, RhoB and RhoBTB2. Ubiquitylation was proposed as a mechanism to control the local activity of Rho GTPases, and it can both be selected for either guanosine nucleotide-bound form or affect the substrate regardless of its GTP/GDP-binding state. For example, Rac1 is preferentially ubiquitylated when in the active form (i.e., GTP-bound) and located to the PM [53,54]. Recent results show that a strong, positive correlation exists between Rac1 activity and its level of ubiquitylation, while GDI dissociation does not predispose Rac1 to ubiquitylation [55]. The human genome potentially encodes for ~600 different E3 ligases regulating the ubiquitylation of specific substrate subsets, thus conferring a remarkable versatility and range of different processes modulated. A recent E3 ligase identified as regulating the ubiquitylation of Rac1 and Rac2 in human tumor cells is the HECT domain and ankyrin repeat containing E3 ubiquitin protein ligase 1 (HACE1). This E3 ligase binds selectively to GTP-bound Rac1/2 to promote their conjugation to ubiquitin chains and attenuate their activity. This might explain the coupling of oxidative stress and Rac1/2 dysregulation and the poor prognosis in different contexts where HACE1 mutations are found, such as lung cancer and lymphomas [56]. While ubiquitylation is classically conceived as a major route for the turnover of proteins through the proteasome, the importance of autophagy in the regulated turnover of specific proteins, as opposed to the ubiquitin-proteasome system (UPS), has recently emerged. Intriguingly, different studies support an interplay between them modulating RhoGTPases [57]. The role of ubiquitylation as a modulator of the subcellular sorting of RhoGTPase pools and/or its action on specific compartments has been virtually unexplored; for example, HACE1-dependent ubiquitylation and negative modulation of Rac1/2 is conceived as occurring at the PM [56].

Small Ubiquitin-like Modifiers (SUMO) are small proteins remarkably similar in their structure to ubiquitin, although divergent in their amino acid sequence that is covalently attached to lysine residues in the target protein through an analogous but distinct process and machinery. Four distinct paralogs exist in humans. While recent reports show that various members of the Ras small GTPase family are targeted for SUMO conjugation [58,59], the only Rho GTPase described as SUMOylation substrate is Rac1, whose activity is enhanced upon SUMOylation at its C-terminal region [60]. Indeed, blocking SUMOylation decreases Rac1-dependent breast tumor cell migration and promotes autophagy-mediated cell death [61]. Notably, Rac1 SUMOylation depends specifically on SUMO1, through dynamic conjugation and deconjugation regulated by as yet not fully characterized E3 ligases and SENP proteases on acceptor sites that do not conform to ‘classical’ consensus sequences [62,63]. This fact might bear relevance as paralogs SUMO2 and SUMO3 are considered particularly linked to oxidative and heat shock stress responses. SUMOylation is a means for regulating nucleocytoplasmic shuttling, and a major share of SUMOylation substrates are nuclear proteins; to date, we do not know whether SUMOylated Rac1 pools partition specifically to this or other compartments.

### 3.4. Lipid Modifications and Interactions as Modulators of Rho GTPase Subcellular Compartmentalization and Function

With the exception of RhoBTB1 and RhoBTB2, post-translational modification by lipids was described for all Rho GTPases [64], modulating their location and interaction with specific GEFs, and consequently their feeding into downstream signaling pathways. Among them, prenylation and palmitoylation are particularly relevant.

#### 3.4.1. Prenylation

Prenylation, the modification of proteins by isoprenoids, is known to control the localization and activity of several proteins, with a broad impact on cell regulation [65,66,67]. Most prenylated proteins, among them Rho GTPases, contain the CAAX target motif (where C represents the acceptor cysteine, A is an aliphatic amino acid and X is any terminal amino acid). Prenylation is initiated by the attachment of a 15-carbon (farnesyl) or a 20-carbon (geranylgeranyl) isoprenoid to a Cys residue by a protein farnesyltransferase (FTase) or a protein geranylgeranyltransferase I (GGTase I), respectively [68,69,70]. Prenylated proteins accumulate at endoplasmic reticulum (ER) membranes, where they can then be further processed by Rce1 (Ras-converting enzyme a)-catalyzed endoproteolytic cleavage of the AAX amino acids and Icmt-catalyzed carboxyl methylation of the isoprenylcysteine (Isoprenylcysteine carboxyl methyltransferase).

Prenylation confers hydrophobicity to the C-terminus of GTPases, dramatically increasing their capacity to interact with cellular membranes. The prenylated motif can determine both the specific subcellular compartmentalization of proteins to specific domains of the PM or endomembranes and the activity of the modified protein [71,72,73]. While the role of isoprenylation in the targeting of GTPases to membrane surfaces was extensively characterized, our understanding of the impact of the endoproteolysis and methylation steps on Rho GTPase function is less understood. Different studies suggest that post-prenylation processing does not have a prominent role in the modulation of RhoGTPase-dependent actin cytoskeleton remodeling, and the purpose of Rho GTPases serving as substrates for Rce1 and Icmt—particularly considering that carboxyl methylation is energetically costly and is evolutionarily conserved—is a standing question [74,75].

Interestingly, there is a level of specificity in the attachment of prenyl moieties to small GTPases: whereas the Rho GTPases are mainly geranylgeranylated [76], Ras subfamily members are farnesylated. This specificity for either farnesylation or geranylgeranylation is mainly determined by the last residue of the CAAX sequence: Ser, Met, Glu, Ala or Thr favor farnesylation, whereas Leu or Phe residues promote geranylgeranylation [77].

The type of prenylation in GTPases seems to be critical to define their subcellular sorting. This is highlighted by the example of RhoB, which can be modified by both geranyl and farnesyl groups: geranylgeranylated RhoB localizes to late endosomes, while the farnesylated form is detected predominantly at the PM [78]. Rac1, RhoA and Cdc42 are mainly modified by geranylgeranylation. Classical models propose that this modification targets these small GTPases to the PM, but their potential contribution to their sorting to other subcellular compartments where they are also present remains poorly characterized.

Rho GTPase prenylation also regulates their binding to GDI proteins [18,79]. GDIs contain a hydrophobic pocket that interacts with the GTPase-bound lipid tail and shields it from the membrane, keeping the GTPase in the cytosol. This additional level of regulation for Rho proteins was known for a long time; however, the exact mechanism by which the GTPase–GDI dissociation is regulated is not well understood. For the Rac1–RhoGDI interaction, it was shown that phosphorylation of RhoGDI by the Rac1/Cdc42 effector kinase Pak1 could release Rac1[80,81]. Interestingly, this mechanism appears only relevant for Rac1 and RhoGDI because the same phosphorylation of RhoGDI by Pak1 does not impair its capacity to bind Cdc42 or RhoA, two other prominent members of the Rho family.

Small GTPase prenylation also has an impact on their guanosine nucleotide binding state, and non-prenylated Rho GTPase species exhibit increased GTP binding through mechanisms as yet poorly understood [82,83]. A potential explanation is that non-prenylated GTPases bind RhoGDI less efficiently (see above), favoring their interaction with GEFs as compared with prenylated pools. Supporting this interpretation, knockdown of RhoGDI also increases GTP loading of Rho GTPases [84,85]. Alternatively, non-prenylated RhoGTPases might interact less with a GTPase-activating protein. A key challenge in understanding small GTPase biology resides in clarifying this contradictory regulation between prenylation and guanine nucleotide binding state, onto which characterizing the principles of intracellular compartmentalization can provide insight. A non-prenylated Rac mutant (RacSAAX mutant) displays a dual cytosolic and nucleoplasmic distribution [86,87]. Similarly, prenylation of other proteins, such as a plant ortholog of calmodulin, controls the equilibrium between PM vs. nuclear localization [88]. Of note, nuclear lamins require, apart from nuclear localization signals, a CAAX motif for their anchoring to the inner nuclear membrane, stressing the relevance of prenylation for the appropriate subcellular distribution of the proteome [89].

Because of the pervasiveness as a mechanism to regulate GTPases and the extensive information from the mutational analysis of the CAAX motif across several proteins, prenylation was intensively investigated as a pharmacological target. However, available combinations of inhibitors of farnesyltransferase (for targeting Ras GTPases) and GGTase I (for Rho GTPases) exhibit significant toxicity and thus have limited clinical potential [90,91], although they might prove valuable tools to fully characterize the contextual biological roles of GTPase prenylation.

Statins have emerged as a means to pharmacologically modulate GTPase signaling. Statins inhibit hydroxymethylglutaryl-coenzyme A (HMG-CoA) reductase (HMGCR), the rate-limiting enzyme for mevalonate/cholesterol biosynthesis that catalyzes the conversion of HMG-CoA to mevalonic acid. This step is also required for the synthesis of isoprenoid intermediates, such as farnesylpyrophosphate (FPP) and geranylgeranyl pyrophosphate (GGPP), the precursors needed for GTPase prenylation [92,93]. While their therapeutic use is most frequently aimed at lowering circulating cholesterol levels, statins were also evaluated for cancer therapy. Preclinical studies and clinical trials yielded encouraging results [94], although a more detailed understanding of the precise role of prenylation for RhoGTPase spatial and functional regulation, and the impact of statins therein, is still warranted.

#### 3.4.2. Palmitoylation

While not as common as prenylation of the CAAX box, some Rho family members additionally require the covalent addition of a palmitate acyl chain to the adjacent C-terminal hypervariable domain for appropriate membrane association and regulation [95].

S-palmitoylation is the covalent attachment of a palmitate molecule (16-carbon, saturated fatty acid) to the side chain of Cys residues. This PTM regulates the subcellular localization and trafficking of substrates. It may also directly modulate protein–protein interactions by physically masking binding sites or by forcing a binding site into close membrane proximity, hence reducing availability for protein interaction. Palmitoylation can regulate either retention or anterograde trafficking of proteins at the ER-Golgi interface and towards the endosomal system [96]. N-terminal myristoylation (covalent attachment of a 14-carbon fatty acid) or C-terminal prenylation frequently precedes palmitoylation acceptor sites. As opposed to most lipidation events (N-myristoylation, prenylation, O/N-palmitoylation) which are essentially irreversible, S-palmitoylation (hereafter simplified as ‘palmitoylation’) is a reversible modification sorting signal, allowing proteins to rapidly shuttle between intracellular compartments. Indeed, proteins can cycle between palmitoylated and depalmitoylated states within time scales that range from seconds to hours [97]. Protein palmitoylation is less understood than other lipidations because of the lack of strong consensus sequences at modification sites [98], the scarce structural information on the interactions between palmitoyl-transferases and their substrates and the limited range of available analytical techniques. Understanding how protein palmitoylation influences the function of individual proteins in normal and tumor cells is currently an active field of research [99].

Although some proteins spontaneously autopalmitoylate, most palmitoylation events are catalyzed by the zinc finger DHHC-type-containing (ZDHHC) family of palmitoyl S-transferases (PATs), comprised of 23 distinct proteins in mammals. While PATs can exhibit multiple localizations, ER and Golgi are the prime sites of protein palmitoylation activity in mammalian cells, and three specific ZDHHC enzymes (ZDHHC5, ZDHHC20 and ZDHHC21) are preferentially targeted to the PM [100,101,102]. The opposite reaction, the removal of palmitoyl groups, is catalyzed by acyl-protein thioesterases (APTs) 1/2, belonging to the family of serine hydrolases [103,104]. Interestingly, the full repertoire of thioesterases that depalmitoylate proteins and their specificity and functional impact remain largely uncharacterized [102].

Multiple cancer-associated proteins are palmitoylated. A classic example is provided by the Ras family of small GTPases. In addition to being irreversibly farnesylated at a conserved C-terminal CAAX sequence, H-Ras, N-Ras and the K-Ras4A isoform (but not the K-Ras-4B isoform) are reversibly palmitoylated at one (N-Ras and K-Ras-4A) or two (H-Ras) C- terminal residues. Only the dually lipidated (farnesylated and palmitoylated) forms of H-Ras, N-Ras and K-Ras 4A are properly localized to the PM and capable of transforming cells [105,106,107]. Subsequently, other members of this family displaying cysteines residues proximal to the prenylation site were also analyzed in terms of palmitate incorporation, and soon all three rap2A-C, and R-Ras were found to incorporate this lipid moiety. Among the Rho family of small GTPases, palmitoylation was also described. RhoB is palmitoylated at Cys 189 and 192 upstream of the CAAX box motif [108,109], and we reported the ability of Rac1 to incorporate a palmitic acid at Cys 178 [110]. Interestingly, relative palmitoylation levels strongly differ between proteins, and in the case of Rac1, palmitoylation extent is low as compared to H- or N-Ras, and similar to those reported for R-Ras—a GTPase involved in cell adhesion and spreading, probably exhibiting a rapid turnover consistent with the dynamic regulation of Rac1 activity and trafficking. While all Rac paralogs (Rac1, 2 and 3) contain a conserved Cys residue at position 178, they vary in their downstream amino acid composition, and neither Rac2 nor 3 incorporate palmitic acid efficiently. This confirms that the sequence between Cys 178 and the prenylation sites of Rac proteins determines their palmitoylation efficiency. This correlates with their showing clear differences in subcellular compartmentalization: as opposed to Rac1, Rac 2 and 3 accumulate in the perinuclear region and exhibit little targeting to specific PM subdomains called DRMs (Detergent-Resistant-Membranes). This suggests that Rac1 palmitoylation occurs at the PM and promotes its stable association to DRMs to initiate sustained signaling; the specific PATs involved in Rac1 palmitoylation are currently unknown.

Regarding the two other main members of the Rho family, RhoA is not susceptible to palmitoylation, while the ubiquitously expressed major Cdc42 protein isoform is not palmitoylated, a novel brain-specific alternative splicing variant enriched in dendritic spines was reported as a palmitoylation substrate [111,112,113].

**Figure 2:**
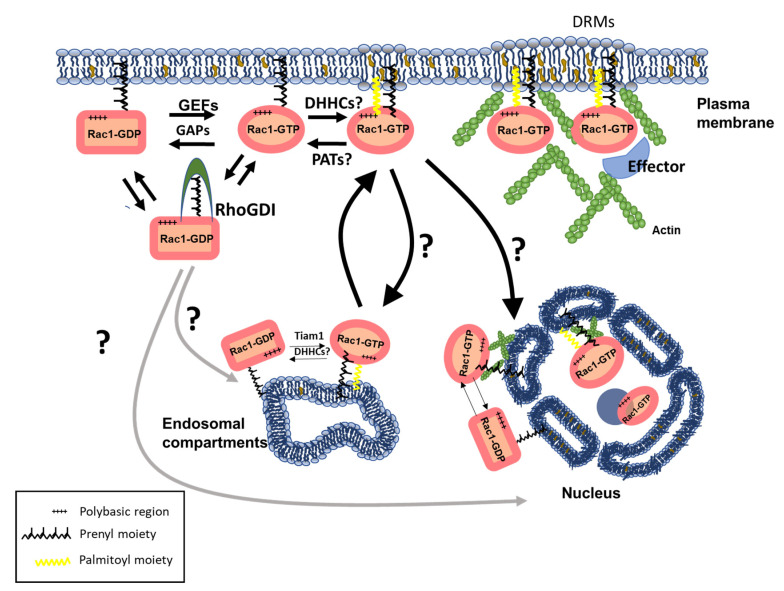
Rac1 trafficking: roles of lipid post-translational modifications in subcellular localization of Rho GTPases, and integration with regulatory mechanisms. The emerging, versatile code embodied by lipid modifications and the regulation they exert on subcellular sorting and activity of substrate GTPases is summarized. Specific examples are depicted here for Rac1 (DHHCs: uncharacterized palmitoyltransferase(s); PAT: uncharacterized palmitoyltransferase(s) transferring acyl chains from GTPase). Incompletely characterized steps are highlighted (?).

Although palmitoylation confers a relatively labile anchorage by itself, its combination with other lipid modifications or polybasic domains can significantly enhance protein attachment to the PM and segregation into specific membrane microdomains enriched in cholesterol, known as ‘lipid rafts’ or cholesterol-enriched plasma membrane microdomains (CEMMs); this is exemplified by Rac1 (Figure 2).

Interestingly, the weakness of the association conferred by palmitoylation is an important feature allowing for the dynamic trafficking across different intracellular compartments, such as endosomes. RhoB was the first Rho GTPase described as associating with the endosomal compartment [114]; subsequent studies identified RhoD, Rac1, Cdc42, TCL or TC10. While the precise mechanism for the recruitment of small GTPases to endosomes is poorly understood, palmitoylation is likely a key event [115]. Multiple studies demonstrated the importance of endosomal pools of Rho GTPases for actin-based endocytic vesicle movement. Endosomal Rac1 was described in different eukaryotic organisms [116,117,118]; the formation of Rab5-positive early endosomes is a pre-requisite for endosomal recruitment of Rac1 and its GEF Tiam1. This active pool of Rac1 is then delivered to specific PM domains for localized actin cytoskeleton remodeling. An analogous mechanism accounts for the concentration of Cdc42 at the leading edge of astrocytes [119]; the localization of Cdc42 on endosome-like vesicles also required Rab5. These studies provide evidence for endosomes serving as hubs for spatiotemporal Rho GTPase signaling and raise the question of how this mechanism is controlled. Coordinated lipid modification might be the answer, the dynamic and transient nature of palmitoylation being a key regulatory mechanism for sorting and on/off switching of Rho GTPases across internal compartments. For example, the farnesylated RhoB pool (PM-localized) is functionally distinct from the geranylgeranylated (endosome-localized) pool. Palmitoylation also controls Ras protein localization to recycling endosomes (RE), a transition stage along with their post-Golgi trafficking to the PM [120]. Precise membrane micro-localization of proteins is likely to have additional relevance for internalization, as specific endocytic retrieval pathways originate from definite PM regions [121]. The role of palmitoylation in endocytic regulation is not restricted to internalization, and it appears to facilitate traffic from recycling endosomes back to the PM [122].

By reinforcing the key role of appropriate palmitoylation for RhoGTPase intracellular regulation, the ‘creation’ of a novel palmitoylation site in Cdc42─by substitution of the Arg 186 residue in position 186 to Cys—sequesters this protein at the Golgi, impairing its shuttling to the PM. This overpalmitoylated form of Cdc42 fails to sustain actin filament polymerization and induces exacerbated pro-inflammatory cytokine production due to increased NF-κB activation [123] (Table 1).

### 3.5. Non-Covalent Lipid Interactions

Together with PTMs and in coordination with their lipidation, GTPases are critically regulated by their non-covalent interaction with specific lipid species across different cellular membranes. Rac1 is able to form spatially segregated nanoclusters at PM domains of distinct lipid composition by selectively associating with phosphatidic acid (PA) and phosphoinositol (3,4,5)-trisphosphate (PIP_3_) over phosphatidylserine, PIP_2_ and other abundant anionic phospholipids, and cholesterol. The biological relevance of this selectivity for PA and PIP3 is evidenced when depleting the PM of PA or PIP_3_, which decreases Rac1 PM binding and nanoclustering [124,125,126]. This binding specificity is mediated by the Rac1 C-terminal membrane anchor, in part through its lipidation state: switching the prenyl group from geranylgeranyl to farnesyl promotes Rac1 selective association to phosphatidylserine rather than PA or PIP_3_; thus, reduced palmitoylation also reduces Rac1 affinity for PA and PIP_3_ [127]. Deciphering the information coded by these dynamic changes might benefit from emerging systematic technologies [128,129], but it is a field we have just started to scratch the surface of. These principles bear a remarkable relevance as an additional regulatory layer susceptible to therapeutic intervention, potentially including through diet.

## 4. Rho GTPases at the Nucleus: Trafficking and Functional Impact

The nuclear envelope (NE) is essential for maintaining the unique biochemical identity of the nucleus. The nucleus is a special membrane-bound compartment: two concentric lipid bilayers, continuous with the ER system―the ONM (outer-nuclear-membrane) and the INM (inner-nuclear-membrane), separated by the perinuclear space (PNS)―and with each other at nuclear pores, act as a strong physical barrier. Despite their similarities in lipid composition, the INM and ONM are biochemically distinct: while the ONM has a certain continuity with the rough endoplasmic reticulum (RER), the INM exhibits a distinct set of integral membrane proteins, which provide docking sites for lamins, chromatin and via interaction with ONM proteins, the cytoskeleton [130,131].

Apart from separating the genome and its associated functions from the cell, the nucleus determines the interaction of cells with their physical environment and their ability to migrate through three-dimensional tissue fiber meshworks. With a distinct rigidity, conferred in large part by the nuclear lamina, and significantly larger dimensions as compared with the surrounding cytoplasm, the nucleus of interphase cells most often defines the limit diameter through which migrating cells can squeeze. The nucleus is also a receptor to both mechanotransduction networks signaling from peripheral structures (PM, cytoskeleton), as well as direct forces through its physical connection with the actin cytoskeleton. As such, the nucleus critically contributes to cell mechanoadaptation. Finally, physical forces can have a variable impact on the integrity of the nucleus and the genome, depending on nuclear deformability, a phenomenon that contributes to tumor cell behavior and evolution.

PTMs regulating small GTPases can regulate the nuclear import and function of many nuclear proteins. Prominent nuclear proteins subjected to prenylation are prelamin A and B type lamins, intermediate filament proteins that polymerize to form the nuclear lamina lining the inner side of the INM. Both are farnesylated and carboxymethylated, but prelamin A is further processed by endoproteolysis to mature lamin A, which lacks the final 18 amino acids, including the modified Cys residue. In cells from patients with Hutchinson–Gilford progeria syndrome, a mutant prelamin A protein, progerin, cannot release its prenylated carboxyl-terminal moiety and therefore remains permanently associated with the nuclear envelope (NE), causing aberrant nuclear morphology. Thus, prenylation is important in the nuclear localization of lamins, but it requires to be properly controlled and removed to ensure cell homeostasis. Palmitoylation can also regulate protein shuttling from the PM to the nucleus. Although palmitoylation was first studied for its impact on protein trafficking through the secretory pathway en route to the PM, several nuclear proteins were identified as targets of palmitoylation. Palmitoylation can modulate the nuclear import of transcription factors [132,133], including all sex steroid receptors (SR), which have highly conserved palmitoylation motifs of nine amino acids: for example, estrogen receptor (ER) α translocates from the PM to the nucleus upon de-S-palmitoylation [134]. Acylation of nuclear factor of activated T cells 5a (NFAT5a) affects its nuclear import and modulates high salt stress-mediated transcriptional activity in mammals [135]. Chromatin remodelers and histones also undergo palmitoylation regulating their activity and nuclear localization, proposing a functional mechanism to modulate the stability of H3/H4 tetramer or modify the interaction between chromatin and the nuclear membrane [132,136]. This strongly suggests that palmitoylation may occur in response to metabolic stress controlling nuclear shuttling, directly coupling the metabolic status of the cell with the regulation of gene expression.

Different species of Rho GTPases localize to this compartment in a regulated manner [86,87,137]. As a rule of thumb, Rho GTPases have a flexible 20 residue C-terminal extension known as the hypervariable region, which provides a binding surface for specific downstream effectors and RhoGDIs [138]. It contains a stretch of adjacent lysine and arginine residues known as the polybasic region (PBRs). This element forms a unique electropositive patch that constitutes a canonical nuclear localization signal (NLS) sequence (K(K/R)X(K/R) for some (such as Rac1) but not all (such as RhoA) GTPases [139]

A fraction of total RhoA localizes to the nucleus at steady-state, and its activity is controlled by the GEF Net1. DNA damage signals such as ionizing radiation (IR) modulate nuclear RhoA, but the specific mechanisms underlying its nucleocytoplasmic shuttling and functional relevance are still being investigated [140]. Recent data support the existence of a nuclear speckle localization signal (NSLS) in RhoA, indicating that additional interactions with specific partners might cooperate with NLSs in the nuclear import and retention of RhoA [141].

Apart from the C-terminal polybasic region, which favors its nuclear translocation [139,142], Rac1 contains two dipeptides TP (residues 108–109) and TP (residues 135–136), which cooperate with Rac1 NLS for its partitioning into nuclear speckles [141]. In general, the nucleocytoplasmic shuttling of Rac1 is a tightly regulated mechanism: nuclear import is mediated by the direct interaction of Rac1 with karyopherin alpha2 [143], while its nuclear export is modulated by its interaction with nucleophosmin-1 and two functional internal nuclear export signals (NES) [86]. Whether palmitoylation–depalmitoylation cycles could also specifically impact nucleocytoplasmic shuttling needs to be addressed. Interestingly, Rac1 cycling in and out of the nucleus depends on cell cycle progression, with increased nuclear Rac1 during the late G2 phase [87]. Additionally, recent studies demonstrate that Rac1-GAP B1-7p-chimaerin inactivates nuclear Rac1, negatively regulating the cell cycle [144]. This might hint at additional opportunities to intervene selectively in nuclear Rac1 activity and function.

While the functional relevance of Rac1 signaling at the NE or inside the nucleus is still incompletely understood, nuclear actin polymerization control appears to be a key downstream output. The formation of distinct nuclear actin structures can be observed upon induction of different forms of cell stress [145,146], but they are not readily visualized by conventional F-actin decoration using phalloidin [147,148,149]. Actin nucleation factors promoting filament formation fall into three major classes: (1) the Arp2/3 (actin-related protein 2/3) complex and nucleation-promoting factors (NPFs), (2) formins and (3) the tandem-monomer-binding nucleators [150]; recent research demonstrates all three types can access the nucleus [151]. Stressing the importance of these actin pools for cell homeostasis, malignant cells frequently exhibit high levels of nuclear actin, a feature contributing to tumor progression that can be phenocopied by knocking down exportin-6 (nuclear export actin modulator) in quiescent cells [152].

Nuclear actin was associated with most nuclear functions, ranging from RNA biogenesis to DNA damage responses and repair. Actin pools are found as a constitutive component of all three types of RNA polymerase (classes I, II and III) [145,151,153] purified spliceosomes and subnuclear RNA metabolism compartments (interchromatin granule clusters or ‘nuclear speckles’) [154] and are required for the spatial positioning of transcriptionally active loci for appropriate RNA maturation and export. Chromatin architecture and remodeling and DNA replication [155] are other functions regulated by nuclear actin dynamics. Thus, aberrant GTPase nuclear signaling in cancer is a potential major direct driver of altered genome integrity and gene expression.

Recent studies demonstrate that pre-mRNA splicing can be modulated by nuclear Rac1 [141]. As an essential step in eukaryotic gene expression, pre-mRNA alternative splicing constitutes an essential mechanism to modulate intracellular localization, enzymatic activity, protein stability and post-translational modification of most proteins. Both the subnuclear compartmentalization of Rac1 to nuclear speckles and its influence on pre-mRNA splicing require its phosphorylation at Thr108. The coupling of the subnuclear localization of Rac1 and its pre-mRNA processing regulatory activity highlights the relevance of the spatial organization of pre-mRNA synthesis and maturation in the nucleus; indeed, the opposition of active transcription sites with nuclear speckles is dependent on nuclear actin dynamics and is required for the appropriate formation of mRNA [156]. Further investigation will help us to understand whether mechanical stimuli and actin polymerization mechanisms can modulate specific compartmentalization of Rac1 into nuclear speckles through regulation of Rac1Thr 108 phosphorylation.

The additional, prominent potential functions for nuclear actin, closely related to the dynamical control of the cytoskeleton by Rho GTPases, are physically scaffolding and conferring plasticity to the cell nucleus. In fact, classic experiments have shown that tugging on integrin adhesion receptors results in distortion of the nucleus, reflecting a physical linkage between the cell surface and the nucleus through the cytoskeleton [157]. Local Rac1-dependent changes in nuclear membrane fluidity and order might be crucial for the deformation of the nucleus required during an invasion. Further, nuclear actin likely regulates the structure of the nucleus and the NE, at least in part, through interactions with lamins, intermediate filament proteins that are a key component of the nucleoskeleton [158]. In vitro studies show that actin binds to the C-terminus of lamin A, through which actin dynamics might influence chromatin organization and nuclear structure [159]. Conversely, both A- and B-type lamins can bundle F-actin *in vitro* [160].

**Figure 3:**
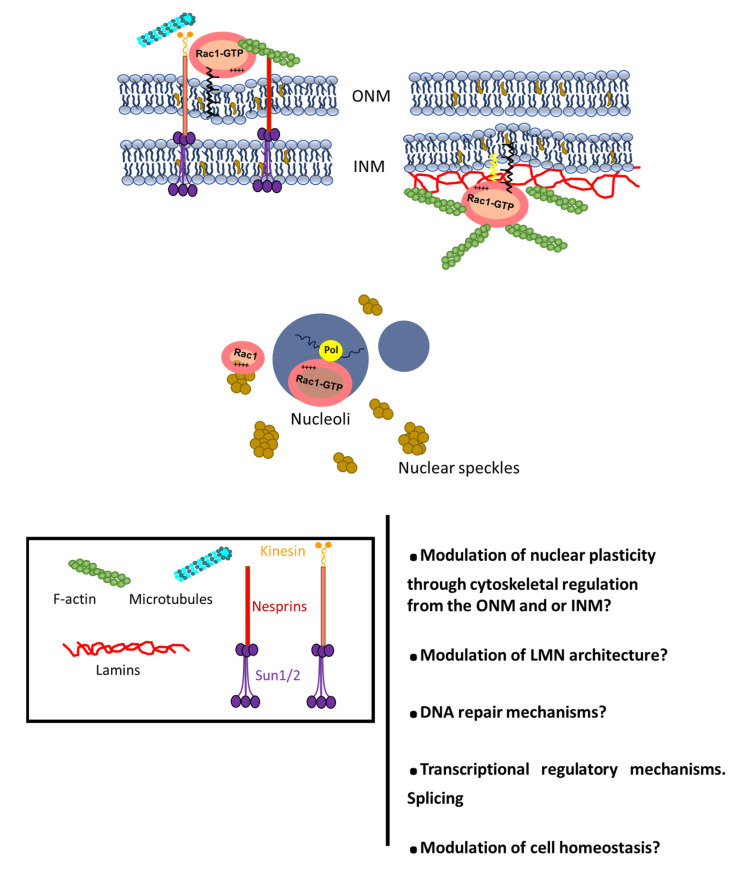
Potential mechanisms for RhoGTPases as modulators of nuclear membrane architecture and organization and nuclear function. ONM: Outer nuclear membrane, INM: Inner nuclear membrane. LMN: Nuclear lamina.

These questions are particularly relevant in the context of genomic integrity. Genotoxic agents activate Rac1 and promote actin polymerization in the nucleus [161,162,163]. Thus, different Rac1 pools might coordinate cell stress responses encompassing DNA repair, genome organization, survival and cell death through the modulation of actin polymerization and different signaling networks (Figure 3). Importantly, cell interaction with its environment, and migration through 3D ECM meshwork and nuclear deformation during metastasis, are sources of mechanical stress, which can itself modulate Rho GTPase activity. Whether mechanical stiffness and architecture of the ECM modulate PTM and nucleocytoplasmic shuttling of RhoGTPases to influence cell migration, proliferation and differentiation is currently an area of intensive research.

Further, the nucleus is subjected to direct mechanical cues and constitutes an emerging mechanotransducer structure in the cell. Different mechanisms determine nuclear mechanics: (i) regulation of lamins, and lamin B receptor expression levels, modulate nuclear envelope integrity and deformability [164]; (ii) the Linker of Nucleoskeleton and Cytoskeleton (LINC) complex is a specialized structure that embodies the early observed physical linkage of nuclear structural components with the cytoskeleton [157,165,166]; (iii) RhoGTPase signaling-dependent actin dynamics might provide a third broad mechanoadaptive component [86,167]. Rac1-regulated actin dynamics contribute to nuclear membrane organization, which in turn can have an impact on LMNA/C-emerin complex distribution, supporting direct crosstalk between nuclear actin network changes and lamin cortex integrity and remodeling; further, Rho GTPase control of the cytoskeleton is an additional input, through the LINC complex, onto nuclear mechanoadaptation. Current investigations demonstrate that Rac1 induces nuclear alterations through microtubules and LINC complex to promote an invasive phenotype in melanoma cells [168]. Rho GTPases are also involved in the specific control of the perinuclear actin cap, a specialized cytoskeletal structure of fibers contacting the nucleus that regulates both nuclear morphology and re-orientation during front-rear polarization. While the detailed mechanisms regulating actin cap dynamics are currently under investigation, recent studies have shown that STEF/Tiam 2, a Rac1-selective GEF that localizes at the nuclear envelope, has the ability to regulate perinuclear Rac1 activity [169] (Figure 3).

Recent studies reveal that mechanical stress can induce conformational changes in (and modulate the PTM of) nuclear envelope proteins [170]. For example, force application results in apical-to basal differences in the conformation of Lamin A/C, as shown by the masking of certain C- and N-terminal epitopes under tension. This suggests that mechanical changes could also directly feed onto PTM and functional modulation of Rho GTPases to control their nuclear compartmentalization and signaling. The specific relevance of these mechanisms, and the principles by which they are coordinated, for pathological processes such as cell transformation and tumor cell aggressiveness, remain an important standing question.

Recent findings reveal how mechanical stimulation by stretching induces an increased expression of RhoA and Rac1[171], with depletion of Rac1 significantly inhibiting cell surface stiffness and 3D migration into extracellular matrices [172]. These data strongly support a bidirectional regulation between mechanical modulation and RhoGTPase function.

An intriguing additional potential layer of coupled regulation arises when considering that nuclear membranes are continuous with the ER. Mechanical stretching of the NE is expected to increase membrane tension at the adjacent RER [173]. Of note, a protein involved in CAAX endoproteolysis of GTPases, Rce1p, is a polytopic transmembrane ER protein; because multiple transmembrane domains are not a common feature of proteases, it may hint at the central importance of ER membrane physical changes in regulating its activity [174,175]. Unfortunately, Rce1p has eluded purification, and neither its amino acid sequence nor its biochemical properties reveal straightforward clues about its mechanisms of action. Inhibitor and bioinformatics analysis suggest that Rce1p may be a serine protease or a metalloprotease, respectively; however, mutagenesis of residues predicted as critical does not affect enzyme activity. The methyltransferase Lcmt is also a multispanning ER membrane protein, with its active site presumably facing the cytosol. The coincidence that both activities involved in the prenylation of RhoGTPases are associated with the ER raises the possibility that mechanical forces can modulate RhoGTPases through ER organization and the regulation of these PTM activities. These associations might also underpin a coupling between lipid metabolism (to which the ER is exquisitely sensitive) [176] and PTM regulation of Rho GTPases. In addition, some of the palmitoyltransferases are also localized in the ER, which can also be modulated by mechanical stretching [177,178].

While Rac1 nuclear expression levels clearly correlate with malignancy, nuclear Cdc42 localization correlates with ER-positive, low-grade tumors [179]. Recent data demonstrate that Cdc42 colocalizes and functionally interacts with components of the endosomal sorting complexes required for transport (ESCRT) machinery—which is in fact required for NE integrity—at NE and ER remodeling sites in yeast [180]. In addition, Cdc42 translocation from the cytoplasm to the nucleus in tumor repopulating cells was reported [181]. Furthermore, nuclear translocation of Cdc42 increases the expression of Tet2, an epigenetic modifier involved in chromatin methylation, strengthening the notion that GTPase-dependent structural and mechanical regulation of the NE feeds into gene expression regulation and genome integrity.

The last aspect worth mentioning regarding nuclear membrane dynamics and the regulation of nuclear pools of Rho GTPases pertains to the control of its lipid composition. Despite the presence of a plethora of biologically active lipids at nuclear membranes and nucleoplasmic structures, such as glycosphingolipids, the main phospholipid component is phosphatidylcholine [182,183], together with other predominantly negatively charged lipids. Cholesterol is also consistently found at the nuclear membrane—albeit at reduced levels, in accordance with its continuity with the ER [184]—where it favors the formation of microdomains; however, recent studies suggest that nuclear membrane lipid ordering or thickness are less variable and less determinant of its properties as compared to membranes strongly enriched in sterols such as the PM (30% and above) [185]. Thus, while nuclear cholesterol could increase stability, the nuclear membrane is nonetheless highly fluid [186]. Beyond the direct regulation of the behavior of nuclear pools of small GTPases, these compositional properties of the nuclear membrane integrate its dynamics with the global metabolic status of the cell.

An additional virtually unexplored field is the relevance of the nuclear localization of different disease-associated GTPase mutants. While activating mutations in KRas, NRas and HRas genes have long been recognized and occur in many types of cancer, genetic dysregulation of Rho family GTPases, such as Rac1 and RhoA, have only recently been characterized as the result of extensive cancer genome-sequencing efforts and are associated with rather specific types of cancer.

Regarding Rac1, the paradigm example we have focused on above, its overexpression has long been reported in many cancers, most frequently correlating with poor prognosis and therapeutic resistance [181,182,183]. Recent findings demonstrate that Rac1-dependent chemoresistance involves the upregulation of glycolytic flux as well as the pentose phosphate pathway, with Rac1 repression as a mechanism to reverse chemoresistance [187]. Overexpression of a specific Rac1 splice variant, termed Rac1b, was described in breast, lung and colorectal cancer. This variant contains an additional stretch of 19 amino acids downstream of the switch II domain, which impairs GTPase hydrolysis activity; its upregulation is associated with NF-κB activation, increased the activity of matrix metalloproteinase-3, epithelial-mesenchymal transition (EMT) and genomic instability [188,189,190]. Although the regulation of both Rac1 and Rac1b is dependent on GAPs, an important differential feature lies in the inability of Rac1b to interact with RhoGDI. As a consequence, most Rac1b remains bound to the plasma membrane and is not sequestered by RhoGDI in the cytoplasm. Differential signaling and functionality of Rac1 and Rac1b in the progression of lung adenocarcinoma was reported [191]. This raises the question as to whether differential post-translational modulation of both variants may explain these differences.

Exosome-sequencing efforts have revealed Rac1 activating mutations in malignant melanoma patients. Most of these mutations affect codon 29, changing Pro to either Ser or, less commonly, Leu, which promotes the quick exchange of GDP to GTP and thus altering signaling output. The 3D structure of Rac1 Pro29Ser in the presence of non-hydrolyzable GTP analogs has shown significant differences in the folding of the switch-I motif, which resembles more closely that of Ras. These mutations represent a typical UV signature and occur in up to 9% of sun-exposed, cutaneous transformations. The downstream effects of these mutations are not fully characterized and likely complex and contextual, beyond the relative accumulation of Rac1-GTP. Expression of PD-L1 is specifically affected by Rac1 Pro29Ser but no other Rac1 mutants [192]. Rac1 Pro29 Ser also abrogates haptotaxis in fibroblasts and modulates invadopodia formation in melanoma cell lines [193]. Additional functionally relevant Rac1 mutations include Ala159 (predominantly in head and neck cancers), Asn92, (melanoma, myeloma and sarcoma), Cys157 and the canonical Gly12 (mainly prostate cancer) and Gly61 (testicular germ cell cancer). Other less common mutations were noted at additional sites such as Cys18, altering Rac1 modulation by ROS and RNS. While the relationship between these Rac1 mutations and clinical outcomes is not yet understood, gene set-enrichment analyses indicate that, in general terms, active Rac1 is frequently associated with dysregulation of immunity-related signatures [194].

Virtually no information exists on what the lipid modification status of each of these mutants is and how they are affected regarding their subcellular partitioning. This information could hold the key to new therapeutic approaches against the disorders these mutations are associated with.

## 5. Conclusions and Perspectives

Rho GTPases govern many essential processes, including cell adhesion, survival, cell cycle and migration, which underlie their contribution to disease and justifies their exploration as important therapeutic targets in cancer and other disorders—particularly in relation to their regulatory activity on cytoskeletal dynamics and PM organization. This perspective was recently expanded by the ability of Rho GTPases to signal from compartments different from the PM, such as endosomes and the nucleus. A deeper understanding of the mechanisms underlying the regulated sorting of RhoGTPases across these compartments is thus warranted because currently available small compounds for the modulation of GTPase activity are limited in their efficacy and safety and do not discriminate between subcellular pools that are profoundly distinct in their function. PTMs of RhoGTPases are critical regulators of their subcellular distribution and functional regulation, but we need to fully characterize their interplay and integration with other cellular processes. The role of RhoGTPases as determinants of nuclear function and mechanoadaptation is one such emerging field, increasingly important to understand tumor cell migration and its link with gene expression reprogramming and genome integrity. In this regard, two main non-exclusive mechanisms were identified: (i) nuclear actin dynamics and its control on nuclear membrane deformability and nuclear function; and (ii) modulation of nuclear architecture through integrin signaling and cytoskeletal components such as perinuclear actin fibers, by virtue of specialized linking structures. Improved insights into the interplay between mechanics and post-translational modifications of RhoGTPases could help us to better understand how their spatiotemporally regulated signaling at distinct compartments occurs.

Furthermore, considering that specialized membranous compartments can handle specific metabolic processes, it is possible that RhoGTPases activity inside these compartments holds the key to the coupling between mechanoadaptation and metabolism. It is plausible that regulation of small GTPase trafficking into the nucleus can be part of a safeguard mechanism that degrades and thereby maintains the appropriate levels of the active form of GTPases with the nucleocytoplasmic shuttling of RhoGTPases as a potential mechanism to regulate cell homeostasis.

An emerging concept is the potential role of GTPases in the control of cellular homeostasis through its implication in autophagic processes at the cytosolic level. The possibility exists that the nuclear pool of small GTPases can also contribute to modulate nuclear homeostasis through the control of a novel and unexplored mechanism such as the nucleophagy, by selectively removing damaged or non-essential nuclear material from the cell. Thus, an altered nucleocytoplasmic shuttling could explain the potential contribution of small nuclear GTPases to tumor progression and invasion. Current and future challenges reside in fully understanding the molecular principles governing these functions and their integration with cell behavior (metabolism, signaling, and mechanical cues) at the systems level.

## Figures and Tables

**Table 1:** Post-translational modifications of RhoA, Rac and Cdc42 GTPases. Cys: Cysteine; Tyr: Tyrosine, Lys: Lysine, Ser: Serine and Thr: Threonine.

RhoGTPases	Post-Translational Modifications
Rac1	Prenylation Cys189 Palmitoylation Cys178 Phosphorylation: Tyr32; Tyr64; Ser71; Thr108 Ubiquitylation: Lys147, Lys166 Sumoylation: PBR domain Glutathionylation Cys18, Cys81, Cys118, Cys157
Rac2	Prenylation Cys189 S-Glutathionylation Cys157
Rac3	Prenylation Cys189 Ubiquitylation Lys166
Cdc42	Prenylation Cys189 Palmitoylation Cys188 Phosphorylation: Tyr32; Tyr64; Ser71 Ubiquitylation: Lys147, Lys166
RhoA	Prenylation Cys190 Phosphorylation: Ser26, Tyr32, Tyr66, Ser188 Ubiquitylation: Lys6, Lys7, Lys135

## Abbreviations

PTMs	Post-translational modifications
GDP/GTP	Guanosine diphosphate and guanosine triphosphate
TC	Tumor Cell
TME	Tumor microenvironment
ECM	Extracellular Matrix
CAFs	Cancer Associated Fibroblasts
NE	Nuclear Envelope
GEFs	Guanine nucleotide Exchange Factors
GAPs	GTPase-Activating Proteins
GDI	Guanine nucleotide Dissociation Inhibitors
EGF	Epithelial Growth Factor
SUMO	Small Ubiquitin-like MOdifiers
FTase	Farnesyltransferase
GGTase I	Geranylgeranyltransferase I
PM	Plasma Membrane
ZDHHC	Zinc finger DHHC
PAT	Palmitoyl-S-Transferase
ER	Endoplasmic Reticulum
RER	Rugous Endoplasmic Reticulum
DRMs	Detergent-Resistant-Membranes
CEMMs	Cholesterol Enriched plasma Membrane Microdomains
RE	Recycling Endosomes
ONM	Outer nuclear membrane
INM	Inner-nuclear-membrane
PNS	Perinuclear Space
NLS	Nuclear Localization Signal
NSLS	Nuclear Speckle Localization Signal
NES	Nuclear Export Signal
LINC	Complex Linker of Nucleoskeleton and Cytoskeleton complex
